# Ultrafast cadmium-zinc-telluride-based renal single-photon emission computed tomography: clinical validation

**DOI:** 10.1007/s00247-023-05682-x

**Published:** 2023-05-12

**Authors:** Matthieu Dietz, Nicolas Jacquet-Francillon, Alexandre Bani Sadr, Boris Collette, Pierre-Yves Mure, Delphine Demède, Géraldine Pina-Jomir, Caroline Moreau-Triby, Bastien Grégoire, Pierre Mouriquand, Marc Janier, Anthime Flaus

**Affiliations:** 1 Service de Médecine Nucléaire, Hospices Civils de Lyon, 59 Bvd Pinel, 69634 Lyon, France; 2 INSERM U1060, CarMeN Laboratory, University of Lyon, Lyon, France; 3 Service de Chirurgie Pédiatrique (Urologique, Thoracique et Transplantation), Groupement Hospitalier Est, Hospices Civils de Lyon, Lyon, France; 4 Lyon Neuroscience Research Center, UMR5292, INSERM U1028/CNRS, Lyon, France

**Keywords:** Children, Diagnostic imaging, Kidney disease, Radionuclide imaging

## Abstract

**Background:**

One of the main limitations of ^99m^technetium-dimercaptosuccinic acid (DMSA) scan is the long acquisition time.

**Objective:**

To evaluate the feasibility of short DMSA scan acquisition times using a cadmium-zinc-telluride-based single-photon emission computed tomography (SPECT) system in children.

**Materials and methods:**

The data of 27 children (median age: 4 years; 16 girls) who underwent DMSA SPECT were retrospectively analyzed. Both planar and SPECT DMSA were performed. SPECT images were analyzed using coronal-simulated planar two-dimensional images. A reduction in SPECT acquisition time was simulated to provide 4 series (SPECT-15 min, SPECT-10 min, SPECT-5 min and SPECT-2.5 min). A direct comparison of the planar and SPECT series was performed, including semi-quantification reproducibility, image quality (mean quality score on a scale of 0 to 2) and inter- and intra-observer reproducibility of the scintigraphic patterns.

**Results:**

The overall image quality score (± standard deviation) was 1.3 (± 0.6) for the planar data set, 1.6 (± 0.5) for the SPECT-15 min data set, 1.4 (± 0.5) for the SPECT-10 min data set, 1.0 (± 0.5) for the SPECT-5 min data set and 0.6 (± 0.6) for the SPECT-2.5 min data set. Median Kappa coefficients for inter-observer agreement between planar and SPECT images were greater than 0.83 for all series and all readers except one reader for the SPECT-2.5 min series (median Kappa coefficient = 0.77).

**Conclusion:**

Shortening SPECT acquisitions to 5 min is feasible with minimal impact on images in terms of quality and reproducibility.

**Graphical Abstract:**

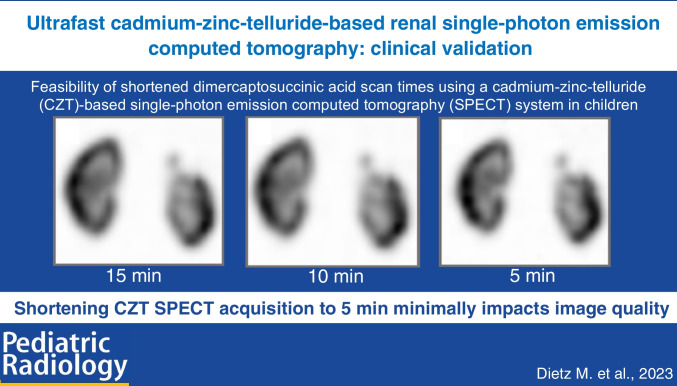

## Introduction

Major advances in nuclear medicine both in the fields of instrumentation and image reconstruction have been reported over the last decade. This progress has translated into shorter scanning time and/or reduced amount of radiopharmaceutical activity administered without loss of image quality compared to conventional techniques. To date, these efforts have focused on positron emission tomography (PET) scans and conventional nuclear medicine, mostly for myocardial investigations [1–3]; little has been done for renal scintigraphy, even though these exams mainly concern pediatric patients who would benefit from shorter scans [4–6].

The^ 99m^technetium-dimercaptosuccinic acid (DMSA) scan is the reference method for imaging the renal cortex and to quantify split renal function [7]. In children, common indications include detection of renal sequelae of pyelonephritis and estimation of functioning renal mass [8]. However, one of the main limitations of DMSA scans is a long acquisition time (15–30 min) [9]. This is particularly important when used in children, who are more prone to move during the acquisition.

Gamma cameras with cadmium-zinc-telluride (CZT) semiconductor detectors have the potential to reduce acquisition times or the injected activity because of their improved contrast and energy resolution [10]. Several studies have shown that CZT single-photon emission computed tomography (SPECT), designed for cardiac imaging, is ultrafast [1]. As such, whole-body SPECT scanning with CZT semiconductor detectors has become commercially available [11]. Although this scanner has the potential to reduce scan times, its clinical efficacy remains to be demonstrated for short DMSA scan acquisition times.

Our hypothesis was that the SPECT CZT gamma camera allows considerable reduction of DMSA scan times, without increasing the radiation dose. The aim of this study was to determine to what extent such reduction is possible in children without losing clinical information.

## Materials and methods

This retrospective study was approved by our institutional review board and informed consent was waived because of its retrospective nature. From October 2016 to March 2017, all children referred for a DMSA scan in our institution were consecutively included.

### Image acquisition

The administered activity was based on patient weight according to the European Association of Nuclear Medicine pediatric dosage card [12]; the median activity was 27 (25–42) MBq (0.73 mCi [0.68–1.14]).

Planar images were acquired 2–3 h after radiopharmaceutical injection using a double head camera Discovery NM/CT CZT 670 (GE Healthcare, Haifa, Israel). Images included anterior, posterior, left anterior oblique, right anterior oblique, left posterior oblique and right posterior oblique projections. Each image required at least 300,000 counts and were reconstructed using a 128 × 128 matrix. In total, planar image acquisition took 30 min per patient.

Immediately thereafter, SPECT was performed using the same gamma-camera. SPECT acquisition, set in body contour mode, comprised 60 projections (30 steps with 6° rotation angle using the dual head gamma detector) of 30 s for an effective 15-min scan time. The raw data were further reconstructed as full-time (30 s/projection: SPECT-15 min) or truncated (20 s/projection, resulting in a simulated 10-min acquisition: SPECT-10 min; 10 s/projection, resulting in a simulated 5-min acquisition: SPECT-5 min and 5 s/projection, resulting in a simulated 2.5-min acquisition: SPECT-2.5 min) time series using Lister software (GE Healthcare). All series were reconstructed and processed under the same conditions using an ordered subset expectation maximization (OSEM: two iterations, 10 subsets), a Butterworth post-filter (0.6 cutoff, factor 10) and a matrix of 128 × 128.

### Semi-quantification and qualitative assessment

SPECT images were analyzed using coronal-simulated planar two-dimensional (D) images. This method is known to be simple and effective for analyzing DMSA SPECT images [13].

Semi-quantitative analyses to evaluate the renal split function were performed on planar and each series of the SPECT images using Xeleris Workstation 3.1™ (GE Healthcare) that automatically placed regions of interest (ROI) for the entire kidney and background noise under visual control of two operators (B.C., a nuclear medicine fellow with 6 months of experience and M.J., a nuclear medicine physician with > 20 years of experience).

**Fig. 1:**
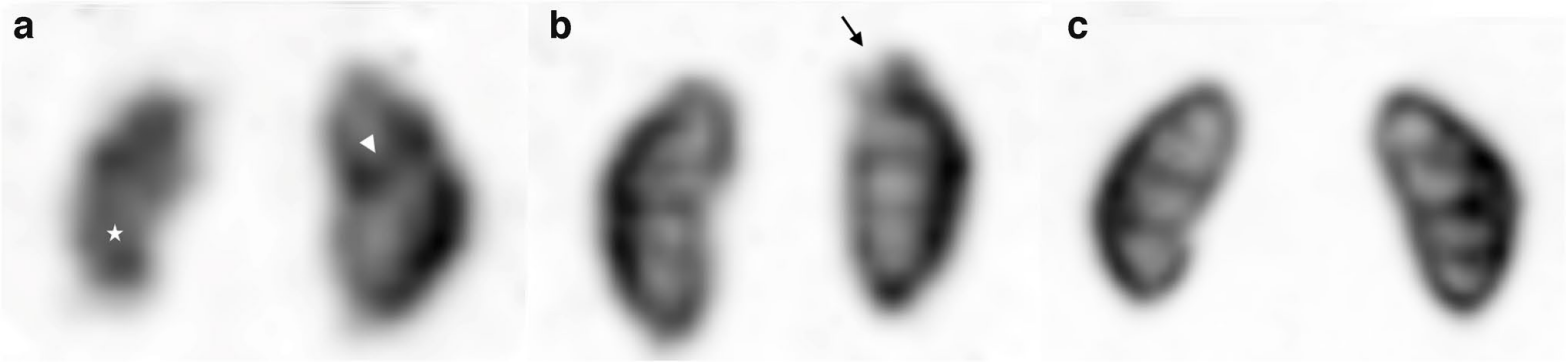
Anterior ^99m^technetium-dimercaptosuccinic acid (DMSA) images of the kidneys illustrating image quality scores. **a** A score of 0 in an 11-month-old girl with bilateral duplex kidneys and recurrent febrile urinary tract infections (UTIs). On the DMSA images, there is evidence of a left duplex kidney (*arrowhead* depicting the cortical bar) and diffuse volume loss of the right lower moiety (*asterisk*) that is probably developmental (reflux nephropathy). **b** A score of 1 in a 7-year-old girl with recurrent UTIs. A small scar is seen in the upper pole of the left kidney (*arrow*). **c** A score of 2 in a 4-year-old boy with recurrent UTIs, who had a normal DMSA scan on presentation

After anonymization and randomization, 3 experienced nuclear medicine physicians (B.G., G.P.J. and C.M.T., with 6, 13 and 12 years of experience, respectively) who regularly read nuclear medicine studies independently reviewed planar and SPECT images, using a grayscale image as recommended [4], blinded to the clinical data and image characteristics. All readers reviewed all the series. A qualitative evaluation of the contrast, resolution and the resulting image quality scores was performed according to the scoring scales and the definitions presented in Table 1. Examples of image quality scores rated as 0, 1 and 2 are presented in Fig. 1. The image quality scores for each and for all reviewers were collected.

**Table 1:** Definitions and scoring scales for the image quality metrics

Metric	Definition	Scoring scale
Resolution	The level of distinguishability between the columns of Bertin and the pyramids	0—“poor” 1—“adequate” 2—“excellent”
Contrast	The contrast between the renal cortex and the medulla	0—“poor” 1—“adequate” 2—“excellent”
Image quality score	The mean of the above two metrics

Inter- and intra-observer agreement of the following scintigraphic findings were evaluated: renal size (symmetrical or asymmetrical) and renal function (symmetrical, decreased right or left renal functioning mass or absent uptake throughout the entire right or left kidney); and for each kidney, contour (preserved or irregular) and cortical uptake (preserved, or single/multiple low or no signal area). To assess intra-observer reproducibility, each series was read twice in random order after a three-month interval.

Continuous data are expressed as mean ± standard deviation (SD) or median [interquartile range] and categorical data as a percentage. The paired sample *t*-test was used to compare the differences in continuous variables between the planar and SPECT series. Bland–Altman plots were generated for each dataset, taking planar images as reference. Inter- and intra-observer reproducibility was assessed using Kappa coefficients. According to Landis and Koch guidelines, Kappa values of 0.21 to 0.40 indicate fair agreement, 0.41 to 0.60 indicate moderate agreement, 0.61 to 0.80 indicate substantial agreement and 0.81 to 1.0 indicate almost perfect or perfect agreement. A statistically significant difference was defined as *P* < 0.05. All statistical analyses were conducted using R version 4.1.1 (R Foundation for Statistical Computing, Vienna, Austria).

## Results

During the study period, 41 patients underwent DMSA scan. Due to technical problems related to the list-mode recording (list-mode data not available, *n* = 5), because of single kidney (non-evaluation of relative renal function, *n* = 2) or due to being 18 years of age or older (n = 7), 14 patients were excluded from subsequent analyses. The remaining 27 children were included in the analysis. The median age was 4 years [0.95–8]; 17 (63%) were 5 years of age or younger; 16 children (59%) were girls and the median body weight was 14 kg [10–26 kg]. No child was sedated.

**Fig. 2:**
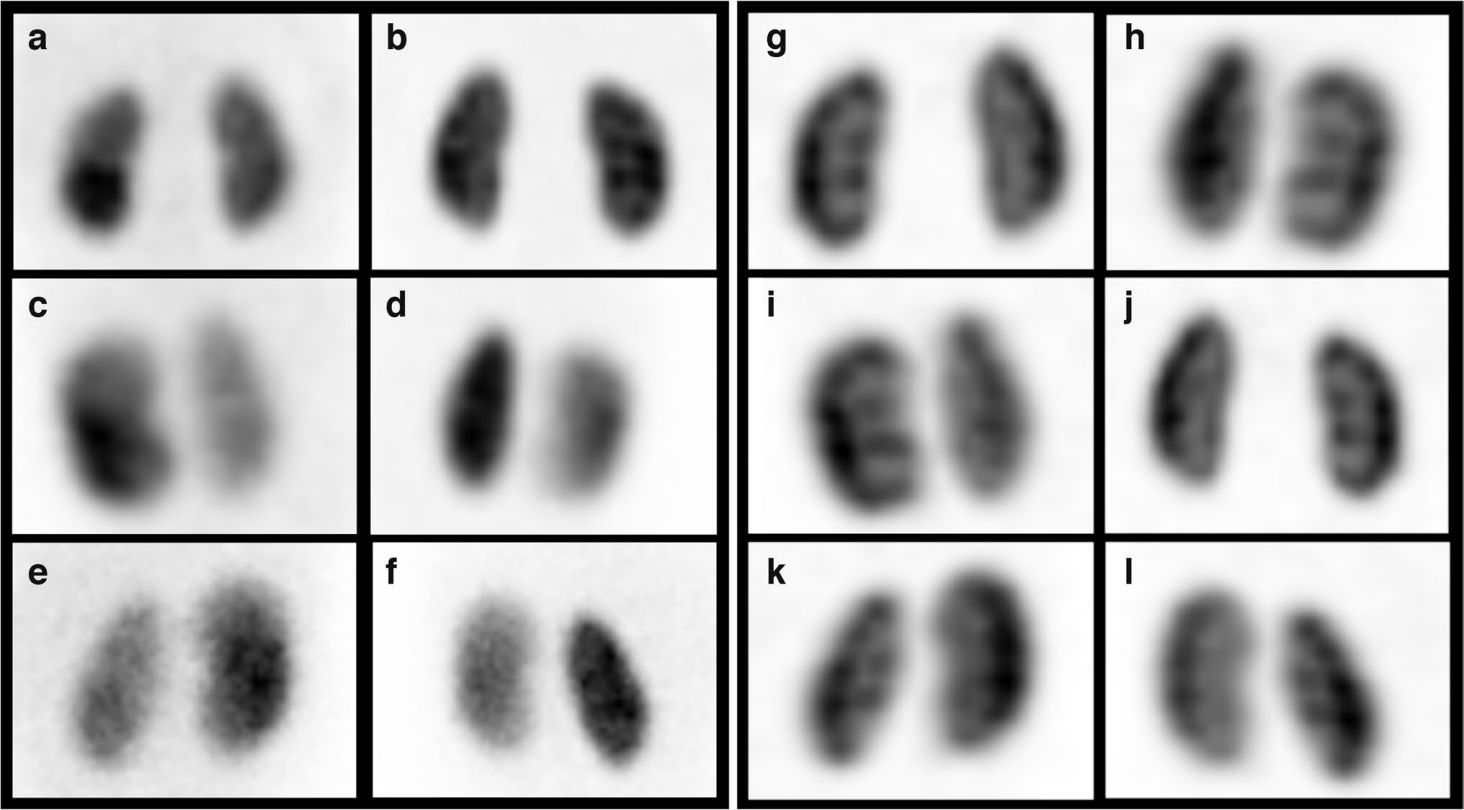
Comparison of planar and single-photon emission computed tomography (SPECT) images in a 1-year-old boy who was referred for a ^99m^technetium-dimercaptosuccinicacid (DMSA) examination to exclude renal parenchymal scarring following an acute urinary tract infection. **a**–**l** Conventional planar images (**a**–**f**) and 5-min scan two-dimensional coronal projection SPECT images (**g**–**l**), showing homogeneous and diffuse cortical renal absorption. Renal function was estimated to be 52% and 48% for the right and left kidneys, respectively. **a**, **g** Anterior images. **b**, **h** Posterior images. **c**, **i** Right anterior oblique images. **d**, **j** Left posterior oblique images. **e**, **k** Left anterior oblique images. **f**, **i** Right posterior oblique images

**Fig. 3:**
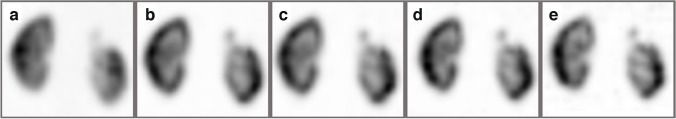
Comparison of planar and single-photon emission computed tomography (SPECT) images in a 3-year-old girl referred for renal scarring 5 months after urinary tract infection with Escherichia coli. Posterior planar image (**a**) and posterior two-dimensional coronal projection SPECT images at 15 min (**b**), 10 min (**c**), 5 min (**d**) and 2.5 min (**e**) showed a scar in the upper pole of the right kidney

Patients were referred for a DMSA scan to exclude renal scars following a febrile urinary tract infection (44%), because of vesico-ureteral reflux (30%) or to estimate relative functioning renal mass (26%). Examples of planar and 2-D coronal projection SPECT images for a boy with a normal DMSA scan (Fig. 2) and for a girl with a renal scar (Fig. 3) are presented.

**Fig. 4:**
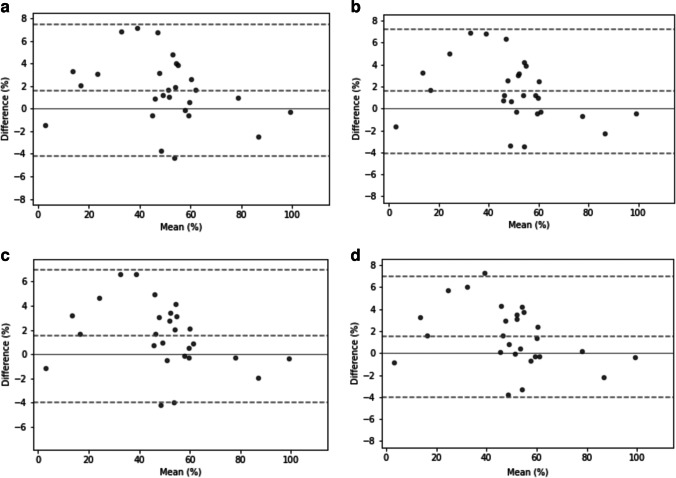
Bland–Altman plots of split renal function comparing planar images to single-photon emission computed tomography images at 2.5 min (**a**), 5 min (**b**), 10 min (**c**) and 15 min (**d**) duration

No significant difference was found between the means of planar and all SPECT series (*P* > 0.7 for all pairwise comparisons). Bland–Altman plots representing agreement of the split renal function estimation between planar and SPECT series are presented in Fig. 4. No mean difference line was significantly different from zero. In SPECT-2.5 min, limits of agreement increased to 7.5 points, compared to 7.2 points for SPECT-5 min and 7.0 points for SPECT-10 min and SPECT-15 min, respectively. Of 27 patients, 1 (4%) had significantly different split renal function measures in all SPECT series as compared to planar series.

The scores for image quality, resolution and contrast gradually improved with an increase in the duration of SPECT acquisition. Results of these evaluations are detailed in Table 2. The overall image quality score was 1.3 (± 0.6) for the planar data set, 1.6 (± 0.5) for the SPECT-15 min data set, 1.4 (± 0.5) for the SPECT-10 min data set, 1.0 (± 0.5) for the SPECT-5 min data set and 0.6 (± 0.6) for the SPECT-2.5 min data set.

**Table 2:** Qualitative assessment

Series		Contrast	Resolution	Image quality scores
Planar	Observer 1	1.7 ± 0.5	1.0 ± 0.2	1.3 ± 0.5
	Observer 2	1.4 ± 0.6	1.3 ± 0.7	1.3 ± 0.6
	Observer 3	1.4 ± 0.5	1.4 ± 0.6	1.4 ± 0.6
SPECT-2.5 min	Observer 1	1.6 ± 0.5	1.1 ± 0.5	0.7 ± 0.6
	Observer 2	0.9 ± 0.7	0.8 ± 0.6	0.7 ± 0.6
	Observer 3	0.9 ± 0.5	0.4 ± 0.6	0.4 ± 0.6
SPECT-5 min	Observer 1	1.8 ± 0.4	1.5 ± 0.6	1.3 ± 0.5
	Observer 2	1.4 ± 0.5	1.2 ± 0.5	1.1 ± 0.5
	Observer 3	1.0 ± 0.7	0.8 ± 0.7	0.8 ± 0.6
SPECT-10 min	Observer 1	1.9 ± 0.2	1.5 ± 0.5	1.9 ± 0.3
	Observer 2	1.6 ± 0.5	1.4 ± 0.6	1.5 ± 0.5
	Observer 3	1.2 ± 0.6	1.1 ± 0.6	1.1 ± 0.6
SPECT-15 min	Observer 1	1.9 ± 0.2	1.5 ± 0.6	1.8 ± 0.4
	Observer 2	1.7 ± 0.5	1.3 ± 0.6	1.6 ± 0.5
	Observer 3	1.4 ± 0.6	1.3 ± 0.7	1.3 ± 0.6

Mean scores ± standard deviation ranging from 0 to 2

*SPECT* single-photon emission computed tomography

Inter-observer reproducibility between planar and SPECT series results is presented in Table 3. Median Kappa coefficients for inter-observer agreement between planar and SPECT images were greater than 0.83 for all series and all readers except one reader for the SPECT-2.5 min series (median Kappa coefficient = 0.77).

**Table 3:** Inter-observer reproducibility between planar and single-photon emission computed tomography (SPECT) series

		SPECT-2.5 min			SPECT-5 min			SPECT-10 min			SPECT-15 min		
		Obs. 1	Obs. 2	Obs. 3	Obs. 1	Obs. 2	Obs. 3	Obs. 1	Obs. 2	Obs. 3	Obs. 1	Obs. 2	Obs. 3
Right kidney	Contours	0.92	0.87	0.71	0.95	0.88	0.88	0.95	0.89	0.87	0.97	0.92	0.97
	Cortical uptake	0.89	0.62	0.76	0.92	0.92	0.84	0.92	0.87	0.89	0.97	0.91	0.97
Left kidney	Contours	0.92	0.81	0.75	0.95	0.89	0.86	1.00	0.95	0.92	0.95	0.89	0.95
	Cortical uptake	0.97	0.82	0.77	0.92	0.87	0.81	0.95	0.95	0.87	0.97	0.87	0.97
	Size	0.92	0.84	0.89	0.97	1.00	0.87	0.95	0.92	0.90	0.97	0.92	0.97
	Function	0.95	0.95	0.88	0.95	0.89	0.88	1.00	0.92	0.90	1.00	0.95	1.00
	Median	0.92	0.83	0.77	0.95	0.89	0.87	0.95	0.92	0.90	0.97	0.92	0.97

Kappa coefficients range from 0 to 1

*Obs. *Observer, *SPECT* single-photon emission computed tomography

Results of intra-observer reproducibility are presented in Table 4. Median Kappa coefficients for inter-observer agreement between planar and SPECT images were greater than 0.81 for all series and all readers.

**Table 4:** Intra-observer reproducibility within each series

	Observer 1					Observer 2						Observer 3				
	Planar images	SPECT				Planar images	SPECT					Planar images	SPECT			
		2.5 min	5 min	10 min	15 min		2.5 min	5 min	10 min		15 min		2.5 min	5 min	10 min	15 min
Contours	0.95	0.91	0.93	0.97	0.97	0.90	0.76	0.88		0.92	0.92	0.91	0.75	0.92	0.96	0.92
Cortical uptake	0.95	0.91	0.93	0.97	0.97	0.91	0.78	0.87		0.93	0.92	0.89	0.82	0.91	0.93	0.94
Contours	0.92	0.92	0.95	0.95	0.95	0.92	0.80	0.89		0.94	0.95	0.90	0.77	0.92	0.95	0.92
Cortical uptake	0.92	0.90	0.97	0.97	0.97	0.90	0.82	0.88		0.94	0.93	0.92	0.80	0.90	0.93	0.93
Size	0.95	0.91	0.97	0.97	0.97	0.92	0.88	0.91		0.93	0.95	0.95	0.90	0.93	0.96	0.97
Function	1.00	0.95	0.97	1.00	1.00	0.91	0.90	0.89		0.92	0.95	0.97	0.95	0.95	1.00	1.00
Median	0.95	0.91	0.96	0.97	0.97	0.91	0.81	0.89		0.93	0.94	0.92	0.81	0.92	0.96	0.94

*SPECT* single-photon emission computed tomography

## Discussion

The present study evaluated the feasibility of DMSA scans with short acquisition times using a SPECT CZT gamma camera and the results suggest that DMSA scans can be reduced to a 5-min SPECT acquisition with minimal impact on images in terms of quality and reproducibility. This finding presents an opportunity to significantly reduce DMSA scan times, which could be especially beneficial for pediatric patients. This significant reduction in scan time could provide greater comfort to children, potentially reducing the likelihood of motion artifacts. Regarding the injected dose, this significant reduction in scan time equates to an 83% decrease in administered activity.

In the past few years, SPECT has begun to replace traditional planar scans; this transition has been accelerated largely by advances in the capabilities of new cameras [10]. However, there have only been a few reports on the potential for reducing scan times and/or injection doses using this new SPECT approach. In agreement with the present study, using previous technology with a conventional Anger gamma camera, Sheehy et al. reported the possibility of a substantial reduction of administered activity and/or scanning time [14]. They compared filter back projection and OSEM-3-D reconstruction methods of DMSA SPECT in terms of image quality and radiopharmaceutical administration and concluded that OSEM-3-D was superior and yielded enhanced image quality allowing a 50% potential reduction of administered activity. Lin et al. recently demonstrated that deep learning-based approaches can generate full-acquisition-time DMSA planar images from short-acquisition-time images, thus reducing the acquisition time in pediatric patients [15]. In addition, SPECT combined with low-dose CT has shown promising results in renal parenchymal assessment [5, 6]. However, additional studies are necessary to evaluate the risk–benefit relationship between the added ionizing radiation and the potential benefit of CT correlation. In this study, we used SPECT-based 2-D reconstructions without the use of low-dose CT for attenuation correction and found that a SPECT CZT gamma camera can produce high-quality DMSA SPECT images from a 5-min SPECT acquisition.

The present findings are subject to certain limitations. This study is a retrospective simulation of reduced-count acquisitions with a small sample size and requires prospective validation. In addition, in order to focus on the influence of reduced scanning time, we chose to use the same reconstruction parameters for all SPECT data sets. Therefore, these parameters may not have been optimal for each series and consequently an alteration of resolution and its consequences cannot be excluded because of an insufficient number of algorithmic iterations. Furthermore, whereas 2-D SPECT is known to be simple and effective for visual analysis of DMSA SPECT images [13], it may not be optimal for quantification and it is of note that a recent study reported by Civan et al. suggests that using 3-D measurement methods could be more accurate for pediatric patients [16]. More generally, a significant portion of the children herein were older than 5 years of age, who are more likely to remain still during the examination and the reproducibility between readers could have been overstated using a binary score for the evaluation of renal size (symmetrical or asymmetrical) and renal contour (preserved or irregular) instead of more quantified measures. Finally, the qualitative assessment was conducted by readers from the same institution and therefore this may differ with readers from different institutions.

## Conclusion

Shortening CZT SPECT acquisition time to 5 min is feasible with minimal impact on images in terms of quality and reproducibility.

## Data Availability

The datasets used and/or analyzed during the current study are available from the corresponding author on reasonable request.
